# The Effects of Age, from Young to Middle Adulthood, and Gender on Resting State Functional Connectivity of the Dopaminergic Midbrain

**DOI:** 10.3389/fnhum.2017.00052

**Published:** 2017-02-07

**Authors:** Andrew C. Peterson, Sheng Zhang, Sien Hu, Herta H. Chao, Chiang-shan R. Li

**Affiliations:** ^1^Frank H. Netter MD School of Medicine at Quinnipiac UniversityNorth Haven, CT, USA; ^2^Department of Psychiatry, Yale University School of MedicineNew Haven, CT, USA; ^3^Department of Internal Medicine, Yale University School of MedicineNew Haven, CT, USA; ^4^Veterans Administration Medical CenterWest Haven, CT, USA; ^5^Department of Neuroscience, Yale University School of MedicineNew Haven, CT, USA; ^6^Interdepartmental Neuroscience Program, Yale University School of MedicineNew Haven, CT, USA

**Keywords:** ventral tegmental area, substantia nigra, functional connectivity, aging, sex difference

## Abstract

Dysfunction of the dopaminergic ventral tegmental area (VTA) and substantia nigra pars compacta (SNc) is implicated in psychiatric disorders including attention-deficit/ hyperactivity disorder (ADHD), addiction, schizophrenia and movement disorders such as Parkinson’s disease (PD). Although the prevalence of these disorders varies by age and sex, the underlying neural mechanism is not well understood. The objective of this study was to delineate the distinct resting state functional connectivity (rsFC) of the VTA and SNc and examine the effects of age, from young to middle-adulthood, and sex on the rsFC of these two dopaminergic structures in a data set of 250 healthy adults (18–49 years of age, 104 men). Using blood oxygenation level dependent (BOLD) signals, we correlated the time course of the VTA and SNc to the time courses of all other brain voxels. At a corrected threshold, paired *t*-test showed stronger VTA connectivity to bilateral angular gyrus and superior/middle and orbital frontal regions and stronger SNc connectivity to the insula, thalamus, parahippocampal gyrus (PHG) and amygdala. Compared to women, men showed a stronger VTA/SNc connectivity to the left posterior orbital gyrus. In linear regressions, men but not women showed age-related changes in VTA/SNc connectivity to a number of cortical and cerebellar regions. Supporting shared but also distinct cerebral rsFC of the VTA and SNc and gender differences in age-related changes from young and middle adulthood in VTA/SNc connectivity, these new findings help advance our understanding of the neural bases of many neuropsychiatric illnesses that implicate the dopaminergic systems.

## Introduction

### VTA and SNc Function

As the main dopamine (DA) producing nuclei in the midbrain, the ventral tegmental area (VTA) and substantia nigra pars compacta (SNc) contribute to many cognitive processes, including reward/saliency processing, error prediction for cognitive control and the execution of movement (Haber and Fudge, 1997). Deficits in these dopaminergic functions are implicated in psychiatric disorders such as attention-deficit/hyperactivity disorder (ADHD; Volkow et al., 2007, 2009), addiction (Groman and Jentsch, 2012) and schizophrenia (Lau et al., 2013) as well as movement disorders such as Parkinson’s disease (PD; Braak et al., 2003; Christopher et al., 2015).

Efferent projections from the VTA and SNc are classically divided into mesocortical (VTA → prefrontal cortex [PFC]), mesolimbic (VTA → nucleus accumbens [NAc]) and nigrostriatal (SNc → striatum) systems (Haber and Fudge, 1997). Major inputs to the VTA/SNc are summarized well in a review by Haber and Knutson (2010). Primary inputs come from the ventromedial PFC (vmPFC), orbitofrontal cortex (OFC), dorsal anterior cingulate cortex (dACC), hippocampus and amygdala (Haber and Knutson, 2010). Whereas these may form some of the major pathways to and from the VTA/SNc, the functional networks of the midbrain nuclei are significantly more complex (Düzel et al., 2009; Murty et al., 2014; Tomasi and Volkow, 2014), and important to understanding the etiology of DA-related diseases (Anand et al., 2005; Greicius et al., 2007; Uddin et al., 2008). This study characterizes and compares resting state functional connectivity (rsFC) of the two structures and explores how age and sex affects the connectivities.

The VTA attributes reward value or salience to environmental stimuli to guide behavior towards a desirable outcome (Bromberg-Martin et al., 2010). For example, in response to a reward the medial PFC (mPFC) boosts VTA DA transmission to facilitate attention toward the same rewarding stimulus (Laviolette et al., 2005; Lodge, 2011). Conversely, avoidance responses to a negative stimulus are associated with silencing of VTA DA neurons (Danjo et al., 2014). The VTA thus integrates information on reward/risk, maintains and updates working memory (Hazy et al., 2006), and controls goal-directed behavior (Bromberg-Martin et al., 2010; Redgrave et al., 2010) through reward-based learning (Berridge and Robinson, 1998; Schultz, 2002; Wise, 2004). Thus, we anticipate the VTA to be strongly connected to prefrontal cortical regions.

The SNc is instrumental for action selection and execution of movement (DeLong et al., 1983; Mink, 1996; Haber and Fudge, 1997; Friend and Kravitz, 2014). The SNc is reciprocally connected to the dorsal striatum (DS; Haber and Knutson, 2010). Signals from the DS are carried through the basal ganglia circuit to facilitate (direct pathway) or inhibit (indirect pathway) movement (DeLong et al., 1983; Mink, 1996; Friend and Kravitz, 2014). On the other hand, more recent studies suggested an expanded scope of SNc function. For instance, in monkeys the SNc responds to both rewarding and aversive stimuli, supporting a role for the SNc in mediating responses to saliency (Matsumoto and Hikosaka, 2009). In humans, the dorsolateral midbrain, primarily the SNc, responds the strongest to motivationally salient signals (D’Ardenne et al., 2013) much like the VTA. Thus, the VTA and SNc may not be functionally as distinct as once thought. We expect our study to demonstrate strong SNc connectivity to regions within the basal ganglia, in support of motor function. Additionally, we hypothesize the SNc will demonstrate connectivity to regions within the salience network including the insula. Examining the rsFC may provide a useful venue to unraveling shared and distinct roles of both the VTA and SNc.

### Age-Related Changes in the Dopamine Systems

The cognitive and motor functions that have been attributed to the DA systems are known to decline with age (Morgan et al., 1987; Watanabe, 1987; Kish et al., 1992; Rollo, 2009; Martorana and Koch, 2014; see also Bäckman et al., 2010 for a review). A number of studies demonstrated reduced DA release (Morgan et al., 1987; Kish et al., 1992), as well as reduced DA receptor (Morgan et al., 1987) and DA transporter (Shingai et al., 2014) expression in association with healthy aging (Rollo, 2009; Martorana and Koch, 2014). Age-related decline of DA starts in the third decade of life and continues at a 10% loss each decade after (Bannon and Whitty, 1997; Reeves et al., 2002). Age-related decrease in DA signaling is linked to lower metabolism in frontal and cingulate cortices (Volkow et al., 2000). It is likely that age-related changes in the DA systems will also manifest in functional connectivity. Furthermore, since DA appears to show an inverted U shape relationship with cognitive measures (Cools and D’Esposito, 2011), it is important to know how rsFC of the dopaminergic midbrain may change with age. Tomasi and Volkow (2014) documented distinct changes in VTA and SNc connectivity during child development and suggested that these changes may contribute to reductions in impulsivity during the transition from adolescence to young adulthood. In older adults, selective degeneration of the SNc is known to contribute to the progression of PD (Schulz-Schaeffer, 2015). Given the above-mentioned changes in VTA/SNc functional connectivity from adolescence to young adulthood and the functional implications of changes in the DA system we sought to investigate age-related changes from young to middle adulthood.

### Sex Related Differences in Dopamine Systems

Many neuropsychiatric conditions show sex differences in pathogenesis and clinical manifestation (Byrnes et al., 1999; Croson and Gneezy, 2009; Eaton et al., 2012; Zhou et al., 2014; Beltz et al., 2015; Park and Park, 2016). While women are more likely than men to develop depression and anxiety, men are more likely than women to develop substance use disorders (SUDs; Eaton et al., 2012; Kuhn, 2015). Both mood disorders and SUDs involve DA dysfunction (Goto et al., 2016; Oliva and Wanat, 2016). There are numerous imaging studies characterizing sex differences in regional activations or functional connectivity to task challenges (Li et al., 2009; Gong et al., 2011), some specifically on DA-related functions (Hoeft et al., 2008). For example, computer games are known to increase the release of DA in the NAc (Koepp et al., 1998) and men showed stronger ventral striatal activation in comparison to women during gaming (Hoeft et al., 2008). Likewise, rsFC revealed sex differences in conditions that implicate DA function including ADHD, nicotine dependance, risk-taking behavior (Byrnes et al., 1999; Zhou et al., 2014; Beltz et al., 2015; Park and Park, 2016). Thus, it is important to understand sex differences in the rsFC of the VTA and SNc. If any sex related differences exist within the DA systems, we predict the VTA is more likely to be affected than the SNc since the prevalence of SUDs varies considerably between men and women and the link between the VTA and addiction has been well established.

## Materials and Methods

The study was conducted in accordance with a protocol approved by the Yale Human Investigation Committee.

### Data Set

Resting-state fMRI scans were pooled from three data sets (Leiden_2180/Leiden_2200, Newark, and Beijing_Zang, *n* = 144), downloadable from the 1000 Functional Connectomes Project (Biswal et al., 2010), and our own data (*n* = 106). In selecting the data, we tried to include as many subjects as possible but only datasets acquired under conditions identical to our own (e.g., similar TR, all under 3T, all eyes closed), as in our earlier work (Zhang et al., 2012; Zhang and Li, 2014). Individual subjects’ images were viewed one by one to ensure that the whole brain was covered. A total of 250 healthy subjects’ resting state data (18–49 years of age; 104 men; one scan per participant; duration: 4.5–10 with 8.4 ± 1.6 min) were analyzed. Table 1 summarizes these data sets.

**Table 1:** **Demographic information and imaging parameters of the resting-state functional MRI data obtained from the image repository for the 1000 Functional Connectomes Project and our laboratory**.

Dataset	Subjects	Age (years)	Time points	TR (s)	Slice acquisition order
Beijing_Zang	31 M/66 F	18–26	225	2	Interleaved ascending
Leiden_2180	10 M/0 F	20–27	215	2.18	Sequential descending
Leiden_2200	11 M/8 F	18–28	215	2.2	Sequential descending
Newark	9 M/9 F	21–39	135	2	Interleaved ascending
Our own	63 M/43 F	19–49	295	2	Interleaved ascending

*Note: M, males; F, females; TR, repetition time*.

### Imaging Data Processing

Brain imaging data were preprocessed using Statistical Parametric Mapping (SPM8, Wellcome Department of Imaging Neuroscience, University College London, UK). Images from the first five TRs at the beginning of each trial were discarded to enable the signal to achieve steady-state equilibrium between RF pulsing and relaxation. Standard image preprocessing was performed. Images of each individual subject were first realigned (motion corrected) and corrected for slice timing. A mean functional image volume was constructed for each subject per run from the realigned image volumes. These mean images were co-registered with the high resolution structural image and then segmented for normalization with affine registration followed by nonlinear transformation (Friston et al., 1995; Ashburner and Friston, 1999). The normalization parameters determined for the structure volume were then applied to the corresponding functional image volumes for each subject. Finally, the images were smoothed with a Gaussian kernel of 4 mm at full width at half maximum (FWHM).

Additional preprocessing was applied to reduce spurious blood oxygenation level dependent (BOLD) variances that were unlikely to reflect neuronal activity (Rombouts et al., 2003; Fox et al., 2005; Fair et al., 2007; Fox and Raichle, 2007). The sources of spurious variance were removed through linear regression by including the signal from the ventricular system, white matter, and whole brain, in addition to the six parameters obtained by rigid body head motion correction. First-order derivatives of the whole brain, ventricular and white matter signals were also included in the regression.

Cordes et al. (2001) suggested that BOLD fluctuations below a frequency of 0.1 Hz contribute to regionally specific BOLD correlations. Thus, we applied a temporal band-pass filter (0.009 Hz < *f* < 0.08 Hz) to the time course in order to obtain low-frequency fluctuations, as in previous studies (Lowe et al., 1998; Fox et al., 2005; Fair et al., 2007; Fox and Raichle, 2007).

### Head Motion

As extensively investigated in Van Dijk et al. (2012), micro head motion (>0.1 mm) is an important source of spurious correlations in rsFC analysis. Therefore, we applied a “scrubbing” method (Smyser et al., 2010; Power et al., 2012; Tomasi and Volkow, 2014) to remove time points affected by head motions. Briefly, for every time point t, we computed the framewise displacement given by FD(t) = |Δd_x_(t)| + |Δd_y_(t)| + |Δd_z_(t)| + r|α(t)| + r|β(t)| + r|γ(t)|, where (d_x_, d_y_, d_z_) and (α, β, γ) are the translational and rotational movements, respectively, and r (= 50 mm) is a constant that approximates the mean distance between center of MNI space and the cortex and transform rotations into displacements (Power et al., 2012). The second head movement metric was the root mean square variance (DVARS) of the differences in % BOLD intensity I(t) between consecutive time points across brain voxels, computed as follows: DVARS(t) = $\sqrt{\langle |\text{I(t)}-\text{I(t}-\text{1}){|}^{2}\rangle }$, where the brackets indicate the mean across brain voxels. Finally, to compute each subject’s correlation map, we removed every time point that exceeded the head motion limit FD (t) > 0.5 mm or DVARS(t) > 0.5% (Power et al., 2012; Tomasi and Volkow, 2014). On average, 1% of the time points were removed across subjects.

### Seed Based Correlation and Group Analyses

**Figure 1:**
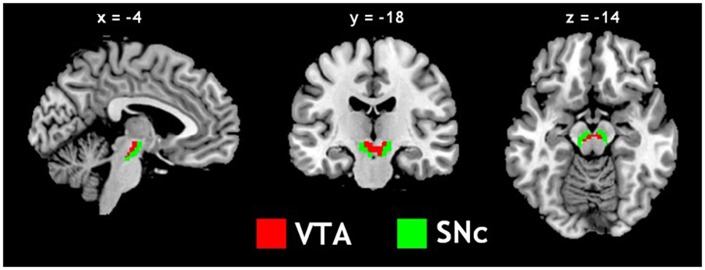
**Seed regions: the ventral tegmental area (VTA) and substantia nigra pars compacta (SNc)**.

The VTA and SNc masks were drawn from 7T MR images (Figure 1) using a region-growing segmentation algorithm (Eapen et al., 2011). This algorithm optimized segmentation and tracing of the VTA and SNc masks to account for intensity inhomogeneities by comparing signal intensity changes from voxel to voxel. Boundaries of the midbrain structures were established according to atlases and the segmentation algorithm was run for every section in the area to produce a 3D-labeled volume map of the VTA and SNc (Eapen et al., 2011). The BOLD time courses were averaged spatially over each of the VTA and SNc seeds. For individual subjects, we computed the correlation coefficient between the averaged time course of each seed region and the time courses of all other brain voxels. To assess and compare rsFC, we converted these image maps, which were not normally distributed, to z score maps by Fisher’s z transform (Jenkins and Watts, 1968; Berry and Mielke, 2000): *z* = 0.5 log_*e*_[(1 + *r*)/(1 − *r*)]. The Z maps were used in group random effect analyses. We performed one-sample *t*-test each on the Z maps of the VTA and SNc and paired-sample *t*-test comparing the two Z maps. A threshold of voxel *p* < 0.05, corrected for family-wise error of multiple comparisons on the basis of Gaussian Random Field theory was used to report the results of one-sample and paired-sample *t*-tests. Whole brain regression analysis with age and two sample *t*-test comparing men and women were performed to examine the effect of age and gender each for VTA and SNc. A threshold combining voxel *p* < 0.001, uncorrected and cluster *p* < 0.05, corrected for family-wise error of multiple comparisons was used to report age and gender findings.

## Results

**Figure 2:**
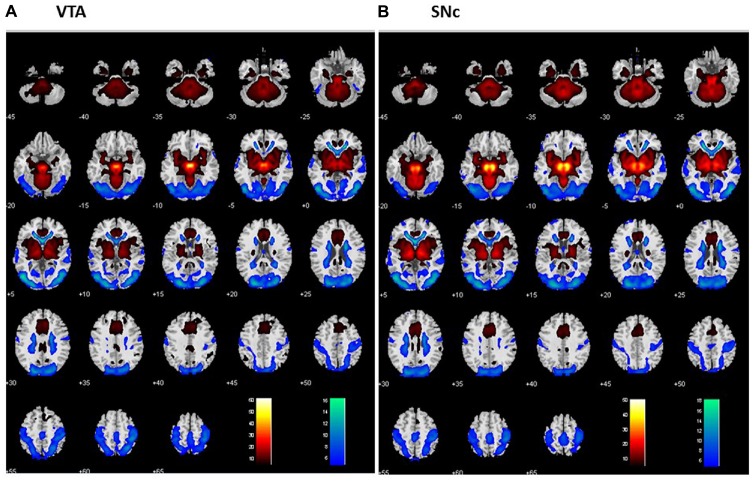
**Whole brain functional connectivity of the VTA (A)** and SNc **(B)** one-sample *t*-test, *p* < 0.05, FWE corrected. Warm color: positive correlation; cool color: negative correlation. Neurological orientation: R = right.

For each seed region, we performed one-sample *t*-test of the Z maps across the group (Figures 2A,B). The VTA and SNc exhibited positive connectivity with the dorsomedial PFC, ventral striatum, thalamus, putamen, pallidum, insula, posterior cingulate cortex (PCC), inferior temporal cortex (ITC), anterior parahippocampal gyrus (PHG), midbrain and large areas of the cerebellum. Both nuclei demonstrated negative connectivity with the occipital cortex, posterior parietal cortex (PPC), precuneus, middle/superior temporal cortex and posterior PHG. These findings replicate earlier rsFC studies of the two dopaminergic structures (Murty et al., 2014; Tomasi and Volkow, 2014; Zhang S. et al., 2016).

### Differences in rsFC of the VTA and SNc

**Figure 3:**
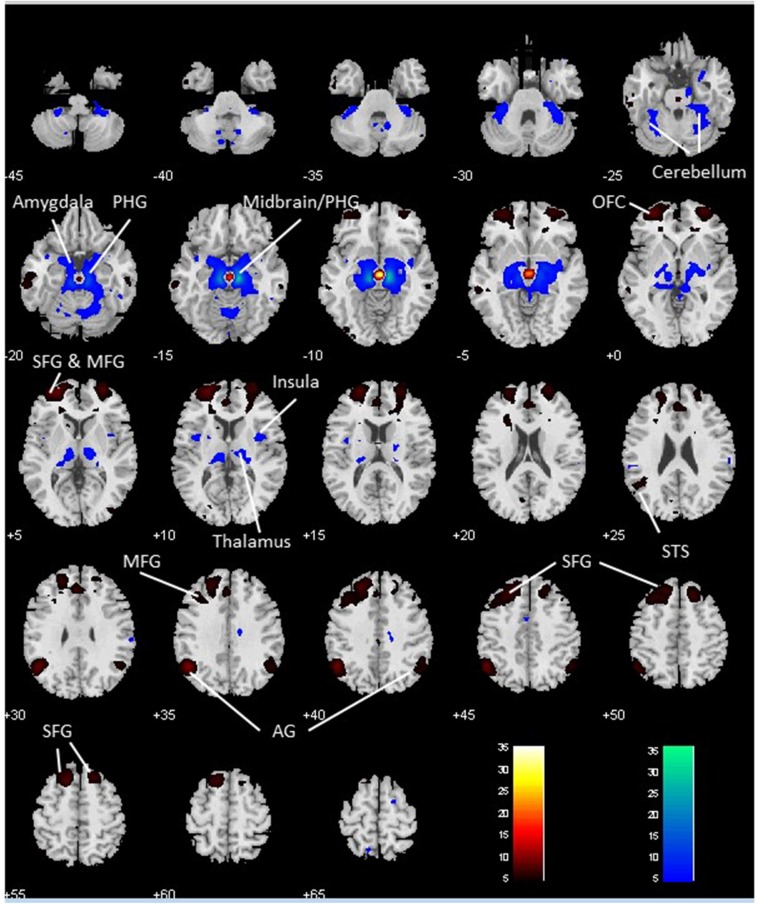
**Differences in whole brain functional connectivity of the VTA vs. SNc: paired-sample *t-*test, *p* < 0.05 FWE corrected.** Warm color: VTA > SNc; cool color: SNc > VTA. Neurological orientation: R, right. AG, angular gyrus; MFG, middle frontal gyrus; OFC, orbitofrontal cortex; PHG, parahippocampal gyrus; SFG, superior frontal gyrus; STS, superior temporal sulcus.

We performed a paired *t*-test to compare functional connectivity of the VTA and SNc (Figure 3). Compared with the VTA, the SNc showed greater connectivity to the thalamus, midbrain, PHG, amygdala, insula and left cerebellum and less connectivity to bilateral angular gyri, left temporal gyrus, bilateral superior frontal gyrus (SFG), ACC and a midbrain region likely including the red nucleus (Table 2).

**Table 2:** **Regions showing differences in functional connectivity to ventral tegmental area (VTA) and to substantia nigra pars compacta (SNc)**.

Volume (mm^3^)	Peak voxel (Z)	MNI coordinate (mm)			Side	Identified brain regions
		*x*	*y*	*z*		
**VTA > SNc**
2349	Inf	3	−19	−11	L/R	Midbrain (Possibly including red nucleus)
7317	Inf	−48	−58	34	L	Angular gyrus/STS
33,831	Inf	−30	56	1	L	SFG/MFG/OFC/ACC
9855	7.49	30	62	4	R	SFG/MFG/OFC
3429	7.21	18	26	52	R	Superior frontal gyrus
2673	6.61	−66	−31	−17	L	Middle temporal gyrus
3321	6.42	48	−55	31	R	Angular gyrus
**SNc > VTA**
46,440	Inf	12	−22	−14	R	Thalamus/Midbrain/PHG/Amygdala/Insula
	Inf	−12	−22	−11	L	Thalamus/Midbrain/PHG/Amygdala/Insula
3645	7.71	−33	−37	−32	L	Cerebellum

*Note: voxel *p* < 0.05, FWE and cluster > 50 voxels; R, right; L, left; ACC: anterior cingulate cortex; MFG, middle frontal gyrus; OFC, orbitofrontal cortex; PHG, parahippocampal gyrus; SFG, superior frontal gyrus; STS, superior temporal sulcus*.

### Age-Related Changes in rsFC of the VTA and SNc

**Figure 4:**
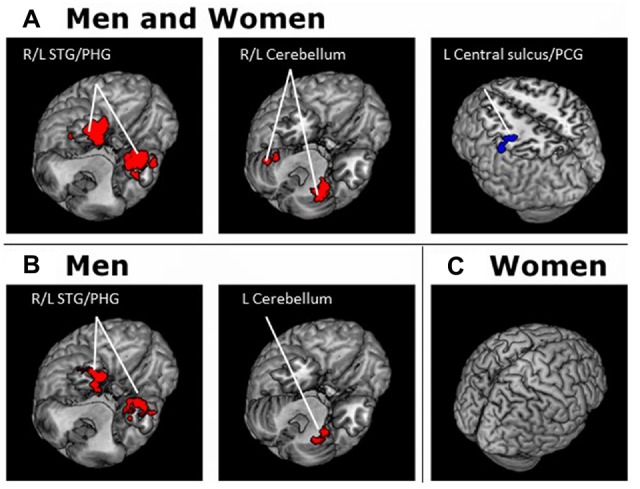
**Brain areas where functional connectivity to the VTA correlates positively (red) and negatively (blue) with age in (A)** men and women combined and in **(B)** men and **(C)** women separately. Voxel-level *p* < 0.001 uncorrected and cluster-level *p* < 0.05, FWE. PCG, Post-central gyrus; PHG, Parahippocampal gyrus; STG, Superior temporal gyrus.

**Figure 5:**
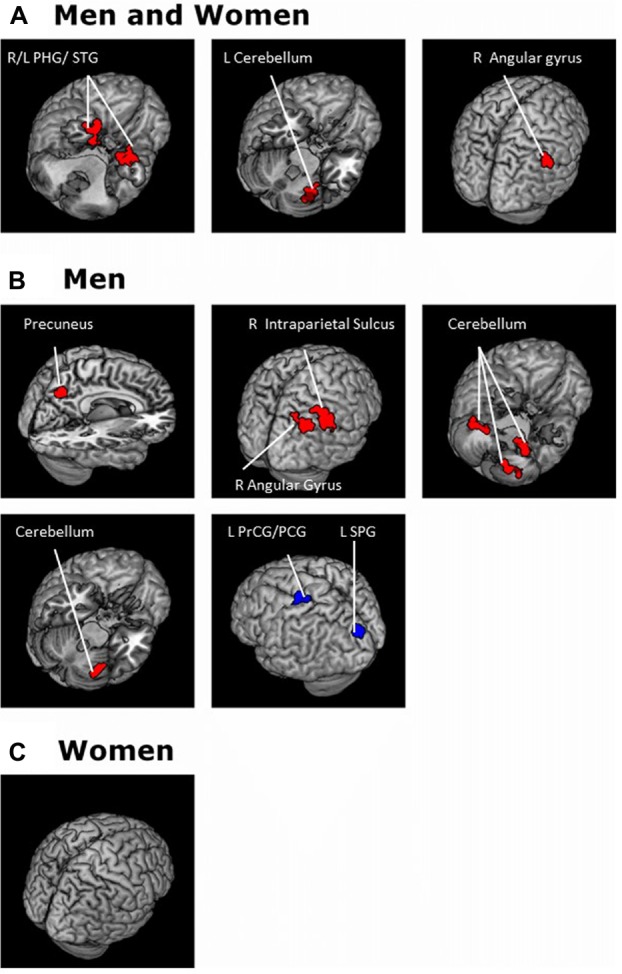
**Brain areas where functional connectivity to the SNc correlates positively (red) and negatively (blue) with age in (A)** men and women combined and in **(B)** men and **(C)** women separately. Voxel-level *p* < 0.001 uncorrected and cluster-level *p* < 0.05, FWE. PCG, Postcentral gyrus; PrCG, Precentral gyrus; PHG, Parahippocampal gyrus; SPG, Superior parietal gyrus; STG, Superior temporal gyrus.

For each seed region we performed a whole brain regression of Z maps against age for the entire group, as well as for men and women separately. For men and women combined, VTA connectivity to bilateral superior temporal gyri, parahippocampus and cerebellum showed positive correlations with age and connectivity to the left central sulcus and postcentral gyrus showed negative correlations with age (Figure 4, Table 3). For men and women combined, the SNc connectivity to the bilateral superior temporal gyrus, bilateral parahippocampus, left cerebellum and right inferior parietal cortex showed positive correlations with age. No negative correlations with age were identified (Figure 5, Table 3). When men were examined separately, VTA connectivity to bilateral superior temporal gyri/parahippocampus and left cerebellum showed positive correlations with age (Figure 4, Table 4), and SNc connectivity to precuneus, right inferior parietal cortex and bilateral cerebellum showed positive correlation with age (Figure 5, Table 4). In men only too, SNc connectivity to the left superior parietal gyrus and left precentral/postcentral gyrus showed negative correlations with age (Figure 5, Table 4). The women only group did not show age-related changes in VTA or SNc connectivities.

**Table 3:** **Regions showing age effects on VTA and SNc connectivity (men and women combined)**.

Volume (mm^3^)	Peak voxel (Z)	MNI coordinate (mm)			Side	Identified brain regions
		*x*	*y*	*z*		
**VTA (positive correlation)**
8073	6.03	30	11	−35	R	Superior temporal gyrus
	4.75	27	5	−29	R	Parahippocampal gyrus (PHG)
6642	5.51	−27	11	−35	L	Superior temporal gyrus/PHG
3780	4.38	−27	−52	−26	R	Cerebellum
1269	4.26	39	−52	−26	L	Cerebellum
**VTA (negative correlation)**
1215	3.89	−39	−22	55	L	Central sulcus
	3.70	−57	−22	49	L	Postcentral gyrus
**SNc (positive correlation)**
1809	5.35	−18	2	−32	L	Superior temporal gyrus/PHG
2808	4.77	27	11	−38	R	Superior temporal gyrus/PHG
2106	4.13	−30	−64	−23	L	Cerebellum
1134	3.79	54	−49	40	R	Angular gyrus
**SNc (negative correlation)**
None

*Note: voxel *p* < 0.001 uncorrected and cluster-level *p* < 0.05, FWE; R, right; L, left; PHG, Parahippocampal Gyrus*.

**Table 4:** **Regions showing age effects on VTA and SNc connectivity for (A) men and (B) women separately**.

Volume (mm^3^)	Peak voxel (Z)	MNI coordinate (mm)			Side	Identified brain regions
		*x*	*y*	*z*		
**A. Men only**
*VTA (positive correlation)*
3510	5.29	−33	−7	−29	L	Superior temporal gyrus/ PHG
4077	4.84	27	11	−38	R	Superior temporal gyrus/ PHG
1890	3.96	−21	−55	−23	L	Cerebellum
*VTA (negative correlation)*
None
*SNc (positive correlation)*
1620	5.60	9	−61	43	L/R	Precuneus/Superior parietal gyrus
4239	4.87	51	−49	49	R	Intraparietal sulcus
1485	4.55	42	−70	43	R	Angular gyrus
1539	4.52	−21	−34	−41	L	Cerebellum
1350	4.34	42	−43	−44	R	Cerebellum
2052	4.13	−24	−67	−47	R	Cerebellum
1053	3.91	−33	−52	−23	L	Cerebellum
*SNc (negative correlation)*
1053	4.39	−38	−91	34	L	Superior parietal gyrus
1404	4.20	−33	−25	70	L	Precentral/Postcentral gyrus
**B. Women only**
None significant

*Note: voxel *p* < 0.001 uncorrected and cluster-level *p* < 0.05, FWE; R, right; L, left. PHG, Parahippocampal Gyrus*.

### Gender Differences in rsFC of the VTA and SNc

**Figure 6:**
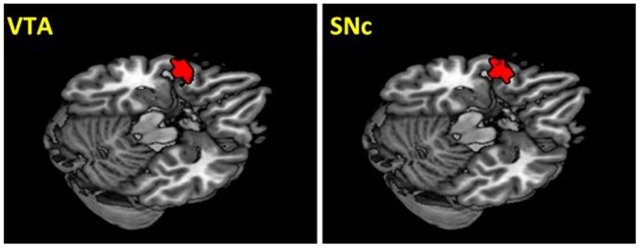
**Brain areas showing differences in rsFC between genders.** Connectivity of both the VTA and SNc to the left posterior orbital frontal gyrus is higher in men than in women. Voxel-level *p* < 0.001 uncorrected and cluster-level *p* < 0.05, FWE.

To compare gender differences in VTA and SNc connectivity we performed a two-sample *t*-test of the Z maps of men and women. Compared to women, men showed greater connectivity of the VTA to the left posterior orbital gyrus (*x* = −33, *y* = 23, *z* = −17; *Z* = 5.12, 1674 mm^3^). Men also showed greater connectivity of the SNc to the left posterior orbital gyrus (*x* = −39, *y* = 20, *z* = −23; *Z* = 4.44, 1296 mm^3^). Compared to men, women did not show higher connectivity in any regions at the same threshold (Figure 6).

In linear regressions, we identified regional connectivity to the VTA and SNc showing age-related changes in men but not women. Thus, we tested for slope difference between men and women in these linear regressions against age for each of the brain regions—three for VTA and nine regions for SNc connectivity (Table 4). The results showed that, at a corrected threshold (*p* < 0.05/12 = 0.004), all except the cerebellum cluster with age-related change in VTA connectivity (−21, −55, −23) demonstrated a significant slope difference (*p*’s < 0.0029, Zar, 1999).

## Discussion

### VTA and SNc Connectivity

We discussed differences in VTA and SNc connectivity, drawing from a broad literature of functional characteristics of the individual brain regions that show differences in VTA and SNc connectivity and of clinical neuroscience of dopaminergic dysfunction.

Compared with the SNc, the VTA showed higher connectivity to cortical structures including the SFG, middle frontal gyrus (MFG) and angular gyrus. Findings of VTA connectivity to frontal structures are consistent with a role of the mesocortical systems in supporting executive function (Haber and Fudge, 1997; Haber and Knutson, 2010). The SFG is involved in cognitive motor control (Li et al., 2013; Hu et al., 2016) and the maintenance (D’Esposito et al., 1998) as well as retrieval (Karlsgodt et al., 2005; Ford et al., 2011) of memory. Methylphenidate, a DA reuptake inhibitor, enhanced activity of the SFG during visual attention and working memory tasks (Tomasi et al., 2011). The MFG, specifically the right MFG, regulates the interaction of goal directed and stimulus-driven attention (Fox et al., 2006). In a patient with right MFG resection, removal of exogenous stimuli was followed by difficulty in reverting back to top-down control (Japee et al., 2015). Thus, greater VTA than SNc connectivity to the SFG and MFG perhaps suggests a more important role of the VTA in dopaminergic regulation of these executive functions.

The angular and temporal gyri are part of the default mode network (DMN; Andrews-Hanna et al., 2014) and VTA connectivity to these regions is consistent with studies implicating dopaminergic regulation of DMN activity (Nagano-Saito et al., 2008; Kelly et al., 2009; Tomasi et al., 2009, 2011) and altered DMN activity in neuropsychiatric illnesses that involve DA dysfunction (Berridge and Robinson, 1998; Roberts and Wallis, 2000; Zago et al., 2008; Volkow et al., 2009; Barbey et al., 2012; Groman and Jentsch, 2012; Lau et al., 2013). Dopaminergic medications such as methylphenidate (Tomasi et al., 2011) and L-DOPA (Kelly et al., 2009) as well as transient DA depletion (Nagano-Saito et al., 2008) alter DMN activity. In another study, DA transporter availability correlated positively with DMN activity during visual attention (Tomasi et al., 2009). In a dimensional change card sorting task the VTA but not the SNc was strongly connected to the cognitive control network during task engagement (Ezekiel et al., 2013). Thus, along with the latter work, the current findings suggest that the VTA but not the SNc may regulate DMN activity and task engagement.

The SNc displayed stronger connectivity to the amygdala, PHG, thalamus, insula and cerebellum in comparison to the VTA, consistent with findings from Tomasi and Volkow (2014) and anatomical projections of the SNc (Carpenter et al., 1976; Beckstead et al., 1979; Zahm and Trimble, 2008; Malenka et al., 2009; Haber and Knutson, 2010). SNc connectivity to the thalamus and cerebellum may support the striatal/cerebellar thalamic cortical circuit for motor control (Haber and Knutson, 2010) and a neural mechanism of tremor in PD (Dirkx et al., 2016). Further, dysfunctional cerebellar cortical circuits have been linked to cognitive and motor impairment in the elderly (Bernard et al., 2013) and DA depletion may contribute to functional deterioration of cerebellar cortical circuits, a pathology associated with increased tremor severity in patients with Holmes’ tumor (Seidel et al., 2009; Kishore et al., 2014). Stronger SNc than VTA connectivity to the cerebellum supports a subcortical mechanism for DA to regulate cerebellar functions.

The insula processes interoceptive information (Gasquoine, 2014) and responds to salient events (Menon and Uddin, 2010; Sterzer and Kleinschmidt, 2010). In Granger causality analysis and structural equation modeling, altered connectivity of the right insula (as in schizophrenia patients) may contribute to multiple network dysfunctions that lead to aberrant salience responses (Moran et al., 2013). In neuronal recordings from behaving monkeys, VTA neurons respond to reward-predicting stimulus only while the SNc neurons respond to both reward and punishment predicting stimuli (Matsumoto and Hikosaka, 2009). Human fMRI demonstrated similar results (D’Ardenne et al., 2013). These findings are consistent with connectivity between the SNc and insula in mediating salience responses irrespective of motivational valence. Of note, impaired interoceptive sensitivity is a non-motor symptom of PD and fatigue in PD is associated with anti-correlated metabolic changes of the saliency network involving the right insula and DMN (Cho et al., 2017).

As discussed earlier, the SNc responds to both positive and negative motivational signals. Thus, stronger positive SNc connectivity to the amygdala suggests a role of the SNc in responding to salient events and perhaps particularly those that predict a negative outcome, furnishing a mechanism whereby the SNc mediates behavioral aversion. The central amygdala projection to the SNc but not VTA may support the encoding of prediction error in appetitive conditioning (Lee et al., 2010). Amygdala hypofunction is postulated to occur with SNc degeneration (Braak et al., 1994), leading to various deficits in emotion processing (Sprengelmeyer et al., 2003) in so-called “amygdala syndrome” in PD (Diederich et al., 2016). In animals, administration of 1-methyl-4-phenyl-1,2,3,6-tetrahydropyridine (MPTP) destroys DA neurons in the SNc and attenuates memory retention in contextual fear conditioning, a process that requires an intact amygdala (Kinoshita et al., 2015). Together, these findings are consistent with a stronger SNc than VTA connectivity to the amgydala and with the relevance of the SNc amygdala circuit to learning and memory.

PHG activity is regulated by DA (Christopher et al., 2015) and critical to the processing of contextual information and memory formation (Diana et al., 2007). Patients with PD exhibit memory impairment along with reduced DAD2 receptor levels in the right PHG (Christopher et al., 2015). The current finding indicates a stronger SNc than VTA connectivity to the PHG, suggesting that degeneration of SNc DA neurons accompanied with disrupted SNc-PHG connectivity may contribute to learning and memory impairment in PD.

### Age-Related Changes in VTA and SNc rsFC

#### Increased VTA and SNc Connectivity to DMN in Older Adults

The DMN is “deactivated” during task performance, in comparison to resting, mind wondering and self-referential processes (Andrews-Hanna et al., 2007; Grady et al., 2010; Roski et al., 2013). With increasing age throughout adulthood, the degree of DMN deactivation is reduced and the DMN and task-positive network (TPN) become less anti-correlated during cognitive engagement (Andrews-Hanna et al., 2007; Grady et al., 2010; Roski et al., 2013). For instance, the DMN is most strongly deactivated during tasks with the magnitude of deactivation corresponding to task difficulty during auditory target detection (Kelly et al., 2008). In an Eriksen flanker task the degree of antiphase correlation between the DMN and TPN was associated with performance consistency (McKiernan et al., 2003). In both studies older adults demonstrate altered DMN activity in association with behavioral decline (McKiernan et al., 2003; Kelly et al., 2008). Here, in support of these earlier findings, we demonstrated weaker negative connectivity of the VTA and SNc to the temporal/PHG—part of the DMN (Andrews-Hanna et al., 2014)—with increasing age.

Transient DA depletion was associated with less deactivation of the temporal lobe during a cognitive task (Nagano-Saito et al., 2008). In contrast, methylphenidate facilitates temporal cortical deactivation during visual attention and working memory, as compared to placebo (Tomasi et al., 2011). There is considerable evidence that DA plays a role in regulating DMN activity (Nagano-Saito et al., 2008; Tomasi et al., 2009, 2011) and that DA systems deteriorate in healthy aging (Morgan et al., 1987; Watanabe, 1987; Kish et al., 1992; Volkow et al., 2000; Rollo, 2009; Bäckman et al., 2010; Martorana and Koch, 2014; Shingai et al., 2014). Therefore, age-related changes of DA signaling and VTA/SNc connectivity may support reduced task-related suppression of DMN activity in older adults.

#### Increased VTA and SNc Connectivity to the Cerebellum in Older Adults

Age-related changes in cerebellar structure and function may contribute to cognitive decline in the elderly (Bernard and Seidler, 2014). Cerebellar motor and cognitive functions are anatomically mediated by projections to motor and non-motor cortical areas via the thalamus (Brodal, 1978; Schmahmann and Pandya, 1997). For instance, our previous study identified a cerbello-thalamo-cortical circuit that supports post-error slowing, a critical component of cognitive control (Ide and Li, 2011). Decreased connectivity in cerebellar cortical networks correlated with age-related decrement in working memory, task switching and visuomotor adaptation (Bernard et al., 2013). In PD, regional homogeneity of the cerebellum is increased compared to healthy controls (Wu et al., 2009). During healthy aging, changes in cerebellar activity was linked to motor performance (Kishore et al., 2014). Remediation of motor function has been demonstrated by cerebellar inhibition during paired associative stimulation (PAS) to induce neural plasticity (Kishore et al., 2014). Enhanced motor cortical response to PAS in the elderly could also be achieved by administration of L-DOPA (Kishore et al., 2014). This suggests that decreased DA may contribute to age-related reductions in motor cortical responsiveness to PAS. Thus, cerebellar activity relates to cognitive and motor performance and the decline of these functions during healthy aging may occur because of DA deficiency (Kishore et al., 2014). In support, methylphenidate reduces VTA/SNc connectivity to the cerebellum (Kline et al., 2016). Together, the findings are in accord with age-related reduction in DA in association with increased VTA/SNc connectivity to the cerebellum.

#### Increased SNc Connectivity to the Right Inferior Parietal Lobule in Older Adults

The right inferior parietal lobule (rIPL) is involved in the detection of salient events and sustaining attention towards task goals (Häger et al., 1998; Clark et al., 2000; Adler et al., 2001; Bunzeck and Düzel, 2006; Singh-Curry and Husain, 2009). Lesions of the rIPL result in attentional impairment (Rueckert and Grafman, 1996, 1998). Other work demonstrated that rIPL activity and connectivity changes with age (Grady et al., 2010; Hu et al., 2012). Thus, age-related changes in SNc-rIPL connectivity may influence saliency processing in older adults. As described earlier, both the SNc and rIPL mediate salience responses (Clark et al., 2000; Bunzeck and Düzel, 2006; Diekhof et al., 2009; Matsumoto and Hikosaka, 2009; D’Ardenne et al., 2013). Older adults directed attention towards a salient stimulus while younger adults only paid attention to contextually relevant information (Schmitt et al., 2015). Indeed, an earlier fMRI study showed that healthy aging is associated with a broad increase in rIPL activity to externally driven events (Hu et al., 2012). Thus, changes in SNc-rIPL connectivity likely contribute to age-related changes in saliency processing.

### Sex Differences in VTA/SNc rsFC

A key sex difference is a stronger rsFC between the VTA and SNc to the left inferior frontal gyrus, pars orbitalis (IFGo, [−33, 23, −17] for VTA and [−39, 20, −23] for SNc) in men vs. women. Studies have linked activations of the left IFGo to response inhibition (Roberts and Wallis, 2000; Swick et al., 2008; Barbey et al., 2012). The OFC responds to reward uncertainty in men performing a gambling task (Abler et al., 2009), a response that appeared to be attenuated by methylphenidate vs. placebo (Schlösser et al., 2009). In an rsFC study examining risk propensity, women demonstrated lower risk propensity along with higher functional connectivity density (FCD) in the left inferior OFC ([−36, 36, −15] and [−42, 36, −18] each for long and short range FCD; Zhou et al., 2014). The authors concluded that connectivity differences of the left inferior OFC contributed to sex-differences in risk propensity. Although not always or directly implicating the OFC in the regulation of risk-taking tendency, other studies have linked DA to decision making during uncertain situations (Schultz et al., 2008). With DA levels chronically augmented by L-DOPA, participants were more likely to engage in risky choices than control subjects (Rutledge et al., 2015), a known side effect of dopaminergic medication treatment of PD (Averbeck et al., 2014). Together, to the extent that we could relate these findings on the IFGo and OFC, stronger VTA/SNc connectivity may represent suppression of orbitofrontal functions and provide a neural mechanism to support greater risk propensity and vulnerability to substance misuse (Kuhn, 2015) in men than women.

### Sex Differences in Age Related Effects

There were sex differences in age-related changes in SNc connectivity to bilateral precuneus, right angular gyrus and right cerebellum. Both the precuneus and angular gyrus are part of the DMN (Andrews-Hanna et al., 2014) and men but not women showed age-related increase in connectivity to the SNc. A previous work of rsFC showed stronger regional homogeneity (ReHo) in the precuneus in men than women (Xu et al., 2015). In contrast to women, men also showed age-related increase in SNc connectivity with the cerebellum. In a previous section, we discussed the role of the cerebellum in motor and cognitive tasks and also the link between cerebellar function and DA. Brain regions that showed age effects in both men and women were for the right and left cerebellum for VTA and left cerebellum only for SNc. Our comparison of sex differences in age-related changes identified an increase in SNc connectivity to the right cerebellum in men but not women. Thus, this difference in SNc-cerebellar connectivity which emerged as a sex effect on age-related changes may represent a subtle distinction in how male and female cerebellar function changes through adulthood (Zhang C. et al., 2016).

### Limitations of the Study and Conclusions

Several limitations should be considered for the study. First, we could not study the functional implications of the current connectivity findings because participants were not assessed for neurocognitive performance. Second, although we reported age-related effects, this sample included only young and middle-aged adults and the findings should be considered specific to this age range. The findings may not generalize to the elderly or clinical conditions that occur primarily in the elderly. Third, although we included multiple data sets in order to have the largest possible sample size, the issue of how site-specific variables, such as different gender ratios across study sites, may impact the current findings need to be considered. In a recent study where we investigated hemispheric lateralization of the rsFC of the ventral striatum, we showed that analyses restricted to our own data set (*n* = 106) were consistently replicated in the Beijing-Zang data set (*n* = 97; Zhang et al., 2017). Fourth, graph theoretical measures may provide new information regarding age-related changes in network properties and may complement findings from the current, seed-based analyses (Zhang C. et al., 2016). Finally, we did not propose specific hypotheses and the study is exploratory. These important issues need to be addressed in future work. In particular, by characterizing the target regions that showed differential connectivity to the VTA and SNc, the findings may facilitate molecular imaging to examine the relationship between rsFC and dopaminergic signaling in the brain.

In summary, we demonstrated both shared and distinct patterns of cerebral functional connectivity of the VTA and SNc. There are also important gender differences in the rsFC of these midbrain nuclei. Age-related changes in the rsFC appeared to be more prominent in men than in women. These findings may add to the systems and clinical neuroscience literature of the dopaminergic system.

## Author Contributions

SZ and CRL contributed to study design. SZ and SH contributed to data collection and analysis. AP, SZ, SH, HHC and CRL contributed to literature review and writing of the manuscript.

## Funding

This study was supported by National Institutes of Health (NIH) grants AA021449, DA026990, DA023248 and K25DA040032, as well as the Peter McManus Charitable Trust. The study was also conducted as a summer fellowship program supported by the Frank H. Netter M.D. School of Medicine at Quinnipiac University. The funding agencies are otherwise not involved in the study or in the decision to publish the findings. We declare no financial interests in the current work.

## Conflict of Interest Statement

The authors declare that the research was conducted in the absence of any commercial or financial relationships that could be construed as a potential conflict of interest.
